# Transverse Testicular Ectopia Presenting as Right Inguinal Hernia in an Adult Patient: An Incidental Finding During Robotic Transabdominal Preperitoneal Repair

**DOI:** 10.7759/cureus.15412

**Published:** 2021-06-03

**Authors:** Farhanul Huda, Bhargav Gajula, Sudhir K Singh, Rajat Piplani, Natasha Choudhary

**Affiliations:** 1 General Surgery, All India Institute of Medical Sciences, Rishikesh, Rishikesh, IND; 2 Paediatric Surgery, All India Institute of Medical Sciences, Rishikesh, Rishikesh, IND

**Keywords:** transverse testicular ectopia, inguinal hernia, unilateral double testis, undescended testis, crossed testicular ectopia, robotic exploration

## Abstract

Transverse testicular ectopia (TTE) is a rare anomaly in which both the testes descend through a single inguinal canal and enter the same hemiscrotum. While TTE most commonly occurs in children, a few cases have been reported in adults as well. In this report, we present a case of TTE found accidentally during robotic exploration for right inguinal hernia with left cryptorchidism. Surgeons who frequently engage in the repair of inguinal hernia should be aware of the diagnostic and management options available to them when this condition is found unexpectedly during exploration.

## Introduction

Transverse testicular ectopia (TTE) or crossed testicular ectopia is synonymously referred to as testicular pseudo duplication, unilateral double testis, or transverse aberrant mal descent, and it involves both the testes descending or migrating through the single inguinal canal to the same hemiscrotum [1]. Other anomalies such as persistent Müllerian duct disorders, inguinal hernias, true/pseudohermaphroditism, and hypospadias are commonly associated with this condition [2]. We discuss a case of TTE that was discovered incidentally during robotic exploration for right inguinal hernia with left-sided undescended testis.

## Case presentation

**Figure 1:**
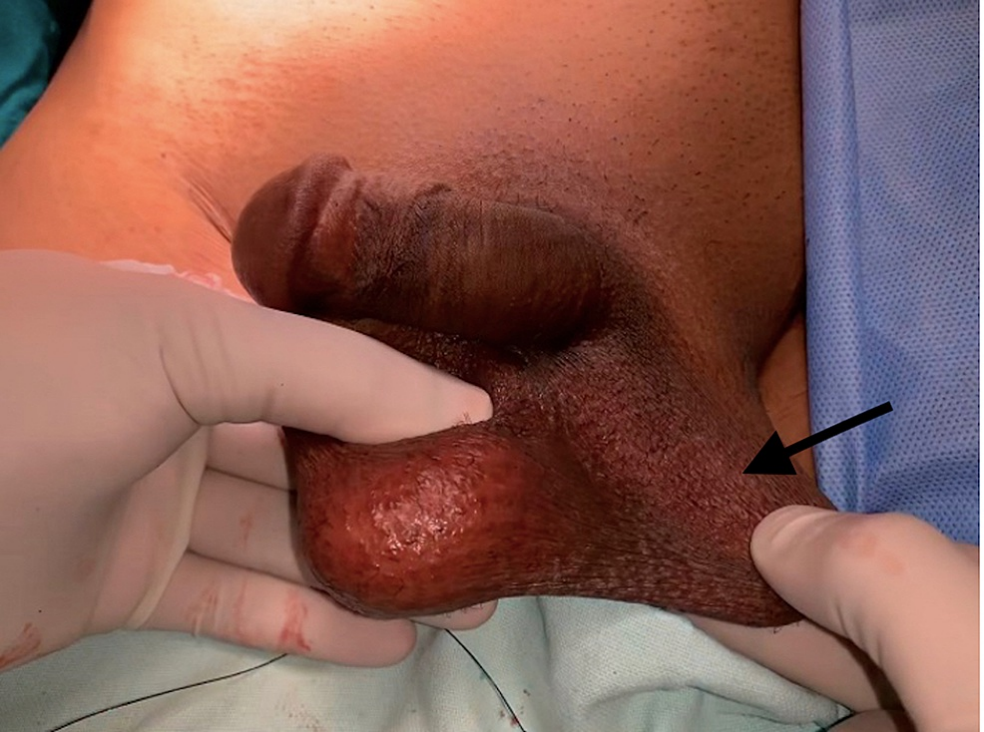
Preoperative clinical picture showing empty left hemiscrotum and reduced right-sided inguinal hernia (black arrow)

**Figure 2:**
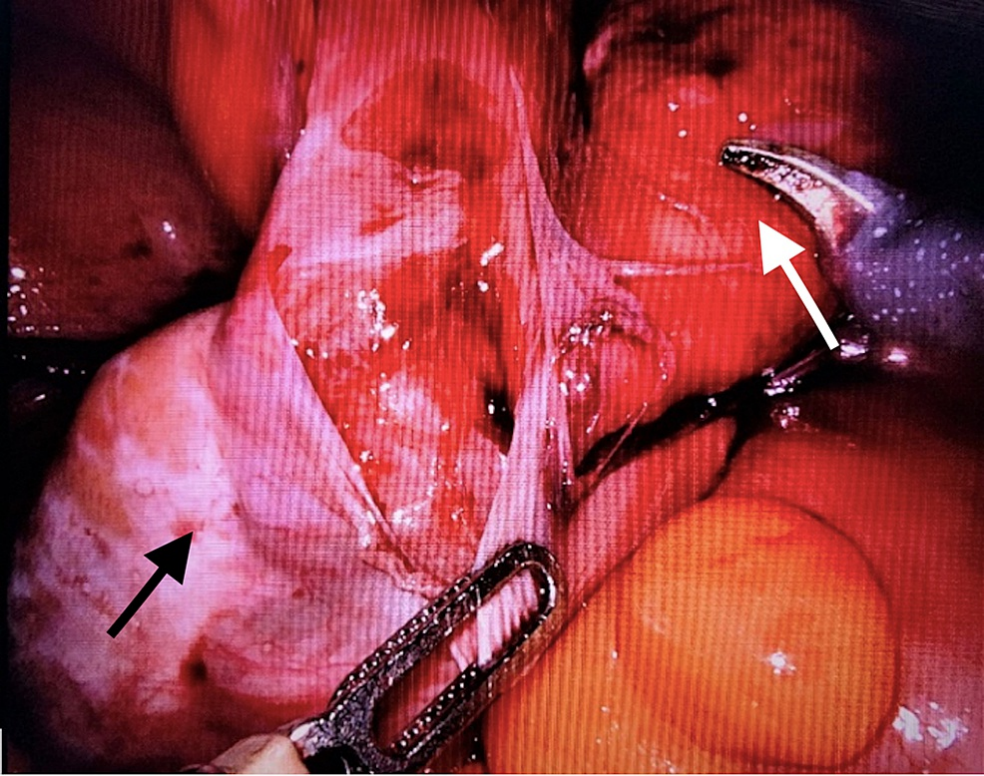
Intraoperative clinical picture showing both the testes in right iliac fossa (white arrow)

A 35-year-old male consulted the surgery outpatient department (OPD) for swelling in the right inguinoscrotal region, which had been first noticed by his mother at 10 years of age. The swelling had been initially small but had gradually increased in size over the last 25 years and had attained the present size of about 8 x 6 cm, which reduced spontaneously when lying down. The patient had no history of chronic cough, constipation, or any surgery in the past. A general physical examination, as well as hematological and biochemical laboratory data, was unremarkable. On local examination, an 8 x 6-cm completely reducible swelling was palpable in the right inguinoscrotal region, extending up to the base of the scrotum. The left hemiscrotum was empty (Figure 1), and a diagnosis of right inguinal hernia with left undescended testis was made clinically; the patient was scheduled for robotic exploration. During the robotic exploration, he was found to have both the separate cords going into the right deep ring. The sac was identified, and on reducing the contents, testis was found in the sac (Figure 2).

**Figure 3:**
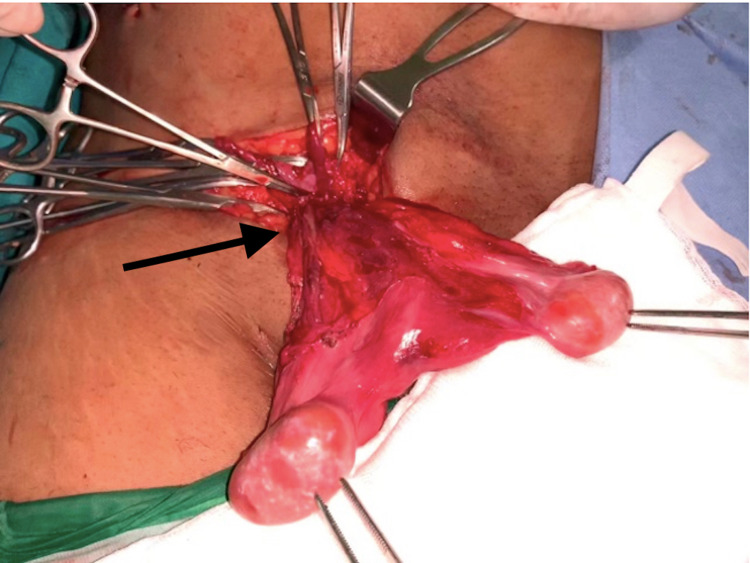
Both the testes with cords delivered through the right inguinal approach (black arrow)

Intraoperatively, a diagnosis of TTE was made. The procedure was then converted to open right mesh hernioplasty with bilateral orchiopexy. A right inguinal incision was made, and bilateral cord structures were released (Figure 3).

**Figure 4:**
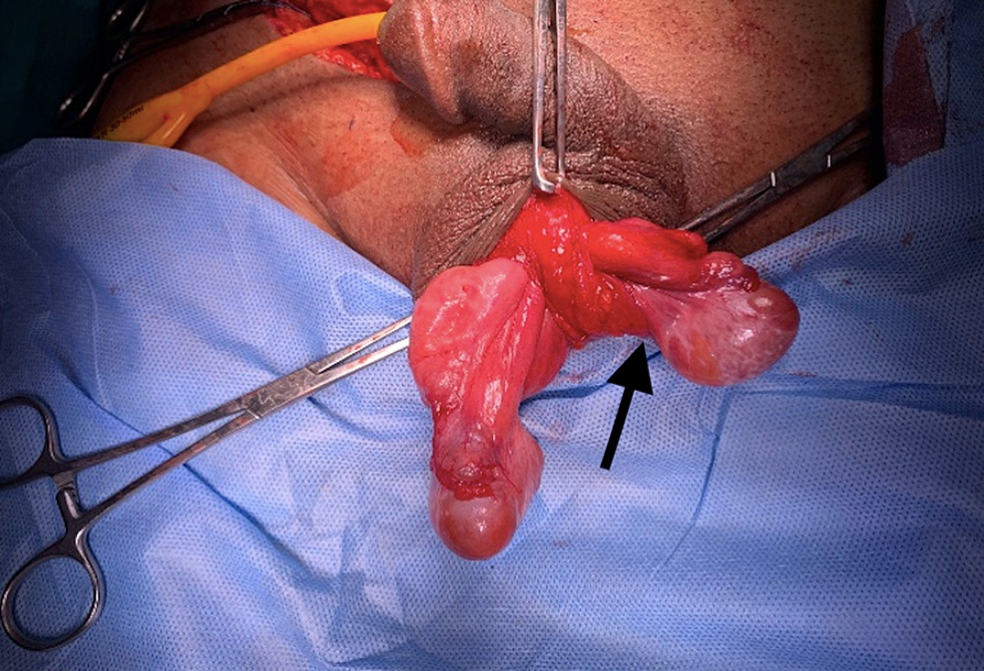
Repositioning of the left testis in its hemiscrotum transseptally (black arrow)

Finally, the left testis, which was smaller compared to the right testis, was transferred transseptally with its cord to the left hemiscrotum and fixed to the sub-dartos pouch (Figure 4).

## Discussion

Von Lenhossek was the first to describe TTE, a rare type of testicular ectopia, in 1886 [1]. Since then, about 100 cases have been reported in the medical literature so far. The pathogenesis of TTE is unclear. However, several theories have been postulated, such as adhesion and fusion of developing Wolffian canals, aberrant gubernaculum, and traction on testis by persistent Müllerian duct syndrome (PMDS). Berg proposed the possibility of the development of both the testes from the same genital ridge [2]. Gupta and Das postulated that due to the early fusion of developing Wolffian ducts, the descent of one testis can cause the second one to follow [3].

PMDS may result from the failure of synthesis or release of Müllerian inhibiting substance (MIS). It has been postulated that the mechanical effect of PMDS prevents testicular descent or leads to TTE [4]. An inguinal hernia is invariably present on the side to which ectopic testis has migrated [5].

TTE has been classified into three groups based on the associated anomalies - type 1: only associated with hernia (40-50%); type 2: associated with PMDS (30%); and type 3: associated with conditions other than PMDS (20%) (hermaphroditism, hypospadias, scrotal anomalies). According to this classification, our case was determined to be type 1 [6].

The mean age of TTE presentation is around four years; it usually presents with cryptorchidism and is often diagnosed intraoperatively during laparoscopic/robotic hernia repair. Cross-over of the vessels and vas of the ectopic testis and the presence of primitive Müllerian duct structures such as the uterus, circular ligament, and fallopian tubes are the most common intraoperative findings [7].

Patients with TTE are at increased risk of malignant transformation. In fact, its overall incidence is about 18% [8]. According to Wood and Elder, performing orchidopexy before the age of 10-12 years reduces the risk of malignancy in undescended testis [9]. Hence, hysterectomy is not routinely recommended in patients who have an obvious uterus and fallopian tubes. Extensive dissection of vas deferens and excision of PMDS should be avoided to prevent injury.

Based on intraabdominal findings, various surgical techniques can be employed. A transseptal orchidopexy is recommended if the vas is of adequate length [10]. Another option is extraperitoneal trans-positional orchidopexy, wherein orthotropic testis can cross transseptally, and ectopic testis with an inadequate length of the vas is placed in the same hemiscrotum.

## Conclusions

TTE is clinically diagnosed based on the presence of two testes in one inguinoscrotal region. TTE should be considered in the differential diagnosis of patients with non-palpable testis and contralateral hernia. Even though preoperative ultrasound of the scrotum or MRI can aid in the diagnosis, diagnostic laparoscopy (DL) can reveal the precise anatomy, detect related abnormalities, and help map out the surgical strategy. It is critical to maintain vascular supply and the position of testes in their hemiscrotum. One must be fully prepared to manage TTE, especially if incidentally diagnosed intraoperatively. Patients with TTE should be followed up in the long term given the increased risk of malignancy.
